# Pathogenesis of Primary Foot-and-Mouth Disease Virus Infection in the Nasopharynx of Vaccinated and Non-Vaccinated Cattle

**DOI:** 10.1371/journal.pone.0143666

**Published:** 2015-11-23

**Authors:** Carolina Stenfeldt, Michael Eschbaumer, Juan M. Pacheco, Steven I. Rekant, Luis L. Rodriguez, Jonathan Arzt

**Affiliations:** 1 Plum Island Animal Disease Center, Foreign Animal Disease Research Unit, Agricultural Research Service, United States Department of Agriculture, Greenport, NY, United States of America; 2 Oak Ridge Institute for Science and Education, PIADC Research Participation Program, Oak Ridge, TN, United States of America; University of Georgia, UNITED STATES

## Abstract

A time-course pathogenesis study was performed to compare and contrast primary foot-and-mouth disease virus (FMDV) infection following simulated-natural (intra-nasopharyngeal) virus exposure of cattle that were non-vaccinated or vaccinated using a recombinant adenovirus-vectored FMDV vaccine. FMDV genome and infectious virus were detected during the initial phase of infection in both categories of animals with consistent predilection for the nasopharyngeal mucosa. A rapid progression of infection with viremia and widespread dissemination of virus occurred in non-vaccinated animals whilst vaccinated cattle were protected from viremia and clinical FMD. Analysis of micro-anatomic distribution of virus during early infection by lasercapture microdissection localized FMDV RNA to follicle-associated epithelium of the nasopharyngeal mucosa in both groups of animals, with concurrent detection of viral genome in nasopharyngeal MALT follicles in vaccinated cattle only. FMDV structural and non-structural proteins were detected in epithelial cells of the nasopharyngeal mucosa by immunomicroscopy 24 hours after inoculation in both non-vaccinated and vaccinated steers. Co-localization of CD11c^+^/MHC II^+^ cells with viral protein occurred early at primary infection sites in vaccinated steers while similar host-virus interactions were observed at later time points in non-vaccinated steers. Additionally, numerous CD8^+^/CD3^-^ host cells, representing presumptive natural killer cells, were observed in association with foci of primary FMDV infection in the nasopharyngeal mucosa of vaccinated steers but were absent in non-vaccinated steers. Immunomicroscopic evidence of an activated antiviral response at primary infection sites of vaccinated cattle was corroborated by a relative induction of interferon -α, -β, -γ and -λ mRNA in micro-dissected samples of nasopharyngeal mucosa. Although vaccination protected cattle from viremia and clinical FMD, there was subclinical infection of epithelial cells of the nasopharyngeal mucosa that could enable shedding and long-term persistence of infectious virus. Additionally, these data indicate different mechanisms within the immediate host response to infection between non-vaccinated and vaccinated cattle.

## Introduction

Foot-and-mouth disease (FMD) is a highly transmissible disease of cloven-hoofed animals caused by foot-and-mouth disease virus (FMDV), an Aphthovirus of the family *Picornaviridae* [1]. The global distribution of FMD is of fundamental concern for international trade in animal products, as countries that are free of the disease (including Europe, North America and Australia) impose strict regulations on import of agricultural products from regions in which the disease is present. Additionally, the disease compromises the health and welfare of livestock and thereby impacts the livelihood of farmers and the consistency of food supply in large regions of the world. Thus, control and prevention of FMD in domestic livestock is critical for all countries that are dependent on agricultural production and trade. Therefore, improving knowledge of the mechanisms of FMDV-host interaction is essential for development of effective intervention strategies, as well as to ensure coherence between applicable countermeasures and official regulatory policies.

Recent investigations of FMDV pathogenesis in cattle have demonstrated that disease progression following controlled aerosol exposure consists of primary infection of epithelial cells in the nasopharyngeal mucosa, followed by dissemination to the lower respiratory tract with subsequent development of viremia and generalized disease [2, 3]. These findings are in agreement with earlier works which also identified the bovine nasopharynx as the anatomic region most susceptible to primary FMDV infection following both intra-nasal instillation and contact exposure [4, 5]. During the clinical phase of infection, the highest quantities of virus are found in the vesicular lesions that are characteristic for the disease and which can be seen within the oral cavity, in inter-digital clefts, on coronary bands and heel bulbs, as well as in other areas of non-haired skin [6, 7]. After resolution of the clinical phase of disease, FMDV is capable of causing a prolonged subclinical persistent infection, which has been reported to occur in approximately 50–100% of infected cattle under experimental conditions [8–13]. Although the mechanisms underlying long-term persistence of FMDV remain incompletely elucidated, most reports have localized the site of virus persistence to the bovine nasopharynx [7, 13–15], thus indicating that this anatomic region has similarly unique roles in early and late stages of FMDV infection in cattle.

Immunization with conventional (inactivated) or recombinant (virus-vectored) FMDV-vaccines that are antigenically matched to the exposure virus protects cattle against clinical FMD [11, 16–20]. However, vaccination generally does not protect animals from infection [5, 11, 16, 17, 21]. Several studies have reported shedding of infectious virus in nasal, oral and/or oropharyngeal fluids by vaccinated animals following virus exposure [5, 16, 19, 20], which is consistent with virus infection of the upper gastrointestinal or respiratory tracts. Additionally, the occurrence of persistent, asymptomatic, FMDV infection in vaccinated cattle [5, 10, 13, 17, 19, 21, 22] provides unequivocal evidence that vaccination does not prevent infection. Thus, subclinical FMDV infection in vaccinated cattle is a conceptually critical event as it enables viral shedding and long-term persistence of infectious virus in animals that have never shown clinical signs of FMD. Yet, to our knowledge, no study has ever documented the specific events that occur during FMDV infection of vaccinated animals.

Recent work in our laboratory has established an optimized, simulated-natural system of direct inoculation of the bovine nasopharynx [23]. Intra-nasopharyngeal (INP) inoculation targets the known sites of primary FMDV infection [2–4] and provides a standardized model that enables precise control of virus challenge, whilst also facilitating natural engagement of the host’s mucosal immune system.

The investigation reported herein includes detailed characterization of the events defining the early phases of FMDV infection following simulated-natural virus exposure of naïve cattle, as well as cattle immunized with a recently licensed adenovirus-vectored FMDV vaccine [24, 25]. These data compare and contrasts infection dynamics and tissue distribution of virus through the initial 96 hours after virus exposure. Further analysis of infected tissues by immuno-microscopy and laser capture microdissection (LCM) provides detailed information on the micro-anatomic distribution of virus and the associated host-response in the two categories of animals.

## Methods

### Animal studies

Animal experiments were performed within the BSL 3-Ag containment facility at the Plum Island Animal Disease Center, New York. All experimental cattle (approximately six month old Holstein steers) were obtained from a certified vendor and were allowed approximately two weeks of acclimation before experiments began.

Sixteen steers (10 non-vaccinated and 6 vaccinated) were used for the studies. Vaccinated and non-vaccinated steers were housed in separate isolation rooms. The investigation presented herein was performed as part of a larger experimental series for which some animals from each treatment group survived for durations up to 35 days post infection (dpi). These surviving animals provided the basis for describing the clinical outcome of FMD infection in each cohort (to be reported in detail in separate publications).

Animals were monitored daily throughout the entire study periods. Experimental procedures were approved by the Plum Island Animal Disease Center Institutional Animal Care and Use Committee (protocol 209-12-R). This experimental protocol delineates defined humane endpoints at which animals are to be euthanized to abrogate suffering. These criteria were not met by any animals included in this study and there were no unexpected animal deaths during the studies. Clinical examinations and inoculations were performed after sedation by Xylazine (0.66 mg/kg) with reversal by intravenous injection of Tolazoline (2.0mg/kg). Non-vaccinated animals that developed marked lameness due to clinical FMD were treated by intramuscular administration of flunixin meglumine (Banamine; 2.2 mg/kg) at 24 hour intervals. All animals were euthanized at pre-determined time points by intravenous injection of an overdose of pentobarbital (90 mg/kg) following sedation by intramuscular injection of Xylazine hydrochloride as described above.

#### Vaccination

Six steers were immunized using a recently licensed recombinant, adenovirus-vectored FMDV serotype A vaccine (Ad5-FMDV; USDA product code 1FM.1R0; manufactured by Antelope Valley Bios, Lincoln, NE). This vaccine construct contains the FMDV A_24_ Cruzeiro, P1-2A and 3C^pro^ coding regions, within a replication deficient human adenovirus vector [24, 25]. Vaccinated steers were intramuscularly injected with the licensed product dose and formulation of 10^7.2^ TCID_50_ Ad5-FMDV with a commercially available adjuvant (ENABL^®^, product number 7010101, VaxLiant, Lincoln, NE, USA). Vaccination was performed 14 days before virus challenge.

#### Challenge virus and inoculation

A bovine-derived FMDV A_24_ Cruzeiro strain with demonstrated virulence in cattle [26] was used for direct inoculation of both vaccinated and non-vaccinated steers. All steers were inoculated with 10^5^ BTID_50_ (50% Bovine Tongue Infectious Doses) of FMDV using a newly optimized simulated-natural system of direct intra-nasopharyngeal (INP) inoculation [23]. In brief, 2 ml of inoculum were deposited within the nasopharynx of sedated steers using a flexible plastic catheter. The length of the catheter was adjusted to the external distance from the nostril to the eye to ensure precise deposition in the nasopharynx based upon previous dissection studies. Studies in vaccinated and non-vaccinated steers were performed in parallel, with the same preparation of inoculum used for both cohorts of animals.

#### Clinical evaluation and antemortem sample collection

Samples collected daily consisted of whole blood in serum separation tubes, as well as oral and nasal swabs from which undiluted secretions were harvested by centrifugation. Swabs were also collected directly after INP inoculation to confirm correct deposition of virus. Two non-vaccinated steers were subjected to more intensive sample collection with oral and nasal swabs collected at 2-hour intervals from 4 to 12 hours post inoculation (hpi). Additional serum samples were collected from these two steers before they were euthanized at 12 hpi. Samples were transported to the laboratory on ice, and were immediately centrifuged for harvesting of serum, nasal secretions and saliva. Sample aliquots were immediately frozen at -70°C until further processing.

All steers were sedated daily for clinical examinations during which oral cavities and feet were thoroughly inspected for signs of FMD vesicles. The progression of clinical infection (lesion distribution) was measured by a semi-quantitative scoring system. In brief, any vesicular lesions observed within the oral cavity (dental pad, tongue, gingival epithelium), lips or nostrils counted for 1 point, with additional points added for vesicles on any of four feet (1 point per foot), giving a maximum of 5 points.

#### Post mortem sample collection

Two non-vaccinated cattle were euthanized by intravenous injection of pentobarbital at each of five pre-determined time points; 12, 24, 48, 72 and 96 hpi (Table 1), with two vaccinated cattle euthanized at each of 24, 48 and 72 hpi (Table 2). As the progression of infection over time was not entirely synchronous across steers within the non-vaccinated group, these animals were aligned by disease progression (clinical disease and viremia) for tabulation and data analyses. Specifically, animals were tabulated by increasing quantitative load of FMDV RNA in blood and grouped into one of three categories: Category I: pre-viremic/pre-clinical (n = 5), Category II: viremic/pre-clinical (n = 2; this category consisted of steers with low level viremia but no clinical signs of FMD at the time of euthanasia), Category III: viremic/clinical (n = 3).

**Table 1:** Tissue distribution of FMDV in non-vaccinated steers.

	Category I					Category II		Category III		
	*(Pre-viremic/ Pre-Clinical)*					*(Viremic/Pre-Clinical)*		*(Viremic/Clinical)*		
*Time point (hpi)*	*12*	*12*	*24*	*48*	*72*	*96*	*24*	*48*	*72*	*96*
*Animal ID*	*1*	*2*	*3*	*4*	*5*	*6*	*7*	*8*	*9*	*10*
***Clinical score***	*0*	*0*	*0*	*0*	*0*	*0*	*0*	*2*	*2*	*4*
***Serum*** *(Log*_*10*_ *GCN/μl)*	neg	Neg	neg	<0.1*	0.35*	**0.38**	**0.46**	**2.95**	**4.94**	**5.37**
***Tissues (*** *Log*_*10*_ *GCN/mg)*										
**Oral cavity/ oropharynx**										
Tongue	1.59		**-**	**-**	**-**	**1.77**	**-**	**+**	**8.87**	**8.14**
Lingual tonsil	**-**	**1.65**	**2.02**	**+**	**+**	**1.57**	1.74	**+**	**5.00**	**3.28**
Palatine tonsil	**-**	**-**	**+**	**+**	**-**	**1.82**	**+**	**+**	**3.84**	**3.88**
Ventral soft palate	**-**	**-**	**2.35**	**2.09**	**2.76**	**2.27**	**1.62**	**-**	**5.95**	**2.77**
**Nasopharynx**										
Dorsal soft palate -Rostral	**-**	**-**	**5.08**	**3.71**	**4.86**	**3.29**	**4.47**	**2.78**	**5.28**	**5.25**
Dorsal soft palate -Caudal	**-**	**2.82**	**6.77**	**3.53**	**2.01**	**3.26**	**6.07**	**4.70**	**5.79**	**4.77**
Dorsal nasopharynx -Rostral	**-**	**3.51**	**5.39**	**3.51**	**4.82**	**3.75**	**3.43**	**4.57**	**4.69**	**5.08**
Dorsal nasopharynx -Caudal	**+**	**3.60**	**2.78**	**2.48**	**3.48**	**4.17**	**1.97**	**3.28**	**4.89**	**4.66**
Lateral nasopharynx -Rostral	**-**	**-**	**+**	**+**	**2.13**	**+**	**+**	**2.87**	**4.27**	**3.34**
Lateral nasopharynx -Caudal	**+**	2.21	**+**	**+**	**+**	**2.73**	**+**	**+**	**5.09**	**1.77**
Nasopharyngeal tonsil	**+**	**+**	**3.15**	**3.94**	**+**	**3.19**	**+**	**3.81**	**1.98**	**+**
**Lungs**										
Proximal cranial lobe	**-**	**-**	**+**	**-**	**-**	**-**	**4.27**	**5.01**	**3.92**	**5.21**
Mid cranial lobe	**-**	**-**	**+**	**-**	**-**	**-**	**3.82**	**2.27**	**4.42**	**4.12**
Distal cranial lobe	**-**	**+**	**-**	**-**	**-**	**-**	**3.51**	**3.10**	**4.71**	**4.85**
Proximal mid lobe	**-**	**+**	**-**	**3.52**	**-**	**-**	**2.84**	**3.63**	**3.20**	**5.39**
Mid mid lobe	**-**	**-**	**-**	**3.05**	**-**	**-**	**2.30**	**2.58**	**3.76**	**3.57**
Distal mid lobe	**-**	**-**	**-**	**-**	**-**	**-**	**1.82**	**2.42**	**4.21**	**4.35**
**Additional tissues**										
Metacarpal skin	**-**	**-**	**-**	**-**	**-**	**-**	**-**	**1.87**	**3.84**	**3.03**
Medial Retropharyngeal LN	**-**	**+**	**+**	**-**	**+**	**+**	**1.64**	**+**	**2.92**	**1.73**
Submandibular LN	**-**	**-**	**-**	**-**	**-**	**+**	**-**	3.99	**2.32**	**3.16**
Hilar LN	**-**	**-**	**-**	**+**	**+**	**-**	**+**	**3.99**	**3.76**	**2.34**
Renal LN	**-**	**-**	**-**	**-**	**-**	**+**	**+**	**+**	**+**	**1.78**
Popliteal LN	**-**	**-**	**-**	**-**	**+**	**-**	**-**	**+**	**2.88**	**3.44**
Interdigital cleft	**-**	**-**	**-**	**-**	**-**	**-**	1.90	**7.51**	**6.56**	**8.52**
Coronary band	**-**	**-**	**-**	**1.72**	**-**	**1.77**	**-**	**+**	**4.00**	**9.02**

Numbers represent log_10_ FMDV genome copy numbers (GCN)/mg of tissue or GCN/μl serum. **Bold** numbers indicate that samples were positive for both FMDV RNA (qRT-PCR) and virus isolation, (+) indicates that virus isolation was positive but FMDV RNA content was below the limit of detection, (-) indicates double negative samples. Limit of detection: 1.53 log_10_ FMDV GCN/mg of tissue, <0.1 log_10_ GCN/ μl serum (corresponding to an assay detection limit of 1.57 log_10_ FMDV GCN/ml serum)

* FMDV RNA detected in sera by qRT-PCR, but samples were negative by VI

**Table 2:** Tissue distribution of FMDV in vaccinated steers.

*Time point (hpi)*	*24*		*48*		*72*	
*Animal ID*	*11*	*12*	*13*	*14*	*15*	*16*
***Clinical score***	***0***	***0***	***0***	***0***	***0***	***0***
*Serum (Log*_*10*_ *GCN/μl)*	neg	neg	neg	neg	neg	neg
*Tissues (Log*_*10*_ *GCN/mg)*						
**Oral cavity/ oropharynx**						
Tongue	**-**	**-**	**-**	**-**	**-**	**-**
Lingual tonsil	**-**	**-**	**-**	**-**	**-**	**+**
Palatine tonsil	**+**	**-**	**-**	**-**	**-**	**+**
Ventral soft palate	**1.66**	**-**	**-**	**-**	**-**	**-**
**Nasal cavity/ Nasopharynx**						
Dorsal soft palate -Rostral	**3.58**	**+**	**3.51**	4.42	**-**	4.04
Dorsal soft palate -Caudal	**4.62**	**+**	**1.78**	2.34	**-**	**+**
Dorsal nasopharynx -Rostral	**4.61**	**+**	**3.74**	**4.88**	**-**	**+**
Dorsal nasopharynx -Caudal	**5.17**	**+**	**-**	3.04	**-**	**4.51**
Lateral nasopharynx -Rostral	**3.35**	**-**	**-**	**-**	**-**	1.76
Lateral nasopharynx -Caudal	**1.75**	**-**	**+**	**-**	**-**	**-**
Nasopharyngeal tonsil	**-**	**+**	**+**	**+**	**-**	**+**
**Lungs**						
Proximal cranial lobe	**-**	**-**	**-**	**-**	**-**	**-**
Mid cranial lobe	**-**	**-**	**-**	**-**	**-**	**-**
Distal cranial lobe	**-**	**-**	**-**	**-**	**-**	**-**
Proximal mid lobe	**-**	**-**	**-**	**-**	**-**	**-**
Mid mid lobe	**-**	**-**	**-**	**-**	**-**	**-**
Distal mid lobe	**-**	**-**	**-**	**-**	**-**	**-**
**Additional tissues**						
Metacarpal skin	**-**	**-**	**-**	**-**	**-**	**-**
Medial Retropharyngeal LN	**+**	**-**	**-**	**-**	**-**	**-**
Submandibular LN	**-**	**-**	**-**	**-**	**-**	**-**
Hilar LN	**+**	**-**	**-**	**-**	**-**	**-**
Renal LN	**-**	**-**	**-**	**-**	**-**	**-**
Popliteal LN	**-**	**-**	**-**	**-**	**-**	**-**
Interdigital cleft	**-**	**-**	**-**	**-**	**-**	**-**
Coronary band	**-**	**-**	**-**	**-**	**-**	**-**

Numbers represent log_10_ FMDV genome copy numbers (GCN)/mg of tissue or GCN/μl serum. **Bold** numbers indicate that samples were positive for both FMDV RNA (qRT-PCR) and virus isolation, (+) indicates that virus isolation was positive but FMDV RNA content was below the limit of detection, (-) indicates double negative samples. Limit of detection: 1.53 log_10_ FMDV GCN/mg of tissue, <0.1 log_10_ GCN/ μl serum (corresponding to an assay detection limit of 1.57 log_10_ FMDV GCN/ml serum)

A standardized necropsy procedure [2] with collection of 25 distinct tissue samples (Tables 1 and 2) was performed immediately after euthanasia. Each tissue sample was divided into aliquots of approximately 30mg which were placed in individual tubes before immediately being frozen on liquid nitrogen. An adjacent specimen was collected from each tissue sample that was divided into two to four replicates, embedded in optimal cutting temperature media (Sakura Finetek, Torrance, CA, USA) in cryomolds, and frozen above a bath of liquid nitrogen. Tissue samples were kept frozen on liquid nitrogen and were transferred to the laboratory within two hours after collection for storage at -70°C until further processing.

Tissue collection was focused upon distinct and clearly defined regions of mucosa- associated lymphoid tissues (MALT) from Waldeyer’s ring, spanning the oro- and nasopharynx [27]. Nasopharyngeal mucosal surfaces were defined as dorsal nasopharynx (nasopharyngeal ceiling), lateral nasopharynx (lateral walls of the nasopharynx, including the tubal tonsil) and the dorsal soft palate (nasopharyngeal floor spanning from the caudal end of the hard palate to the caudal aspect of the soft palate) [2]. Each of these tissues was further subdivided into rostral and caudal segments. Oropharyngeal samples included the ventral soft palate as well as palatine and lingual tonsils [27].

### FMDV RNA detection

Two aliquots of each tissue sample collected at necropsy were individually thawed and macerated using a TissueLyser bead beater (Qiagen, Valencia, CA, USA). 50μl of tissue macerate from each aliquot was transferred to 96-well plates containing 150 ml of lysis/binding solution. RNA was then extracted with Ambion’s MagMax-96 Viral RNA Isolation Kit (Ambion, Austin, TX) on a King Fisher-96 Magnetic Particle Processor (Thermo Scientific, Waltham, MA). RNA was eluted in a final volume of 25 ml. Once extracted, 2.5 ml of RNA was analyzed by quantitative real-time RT-PCR (qRT-PCR), following the protocol described below.

**Fig 1:**
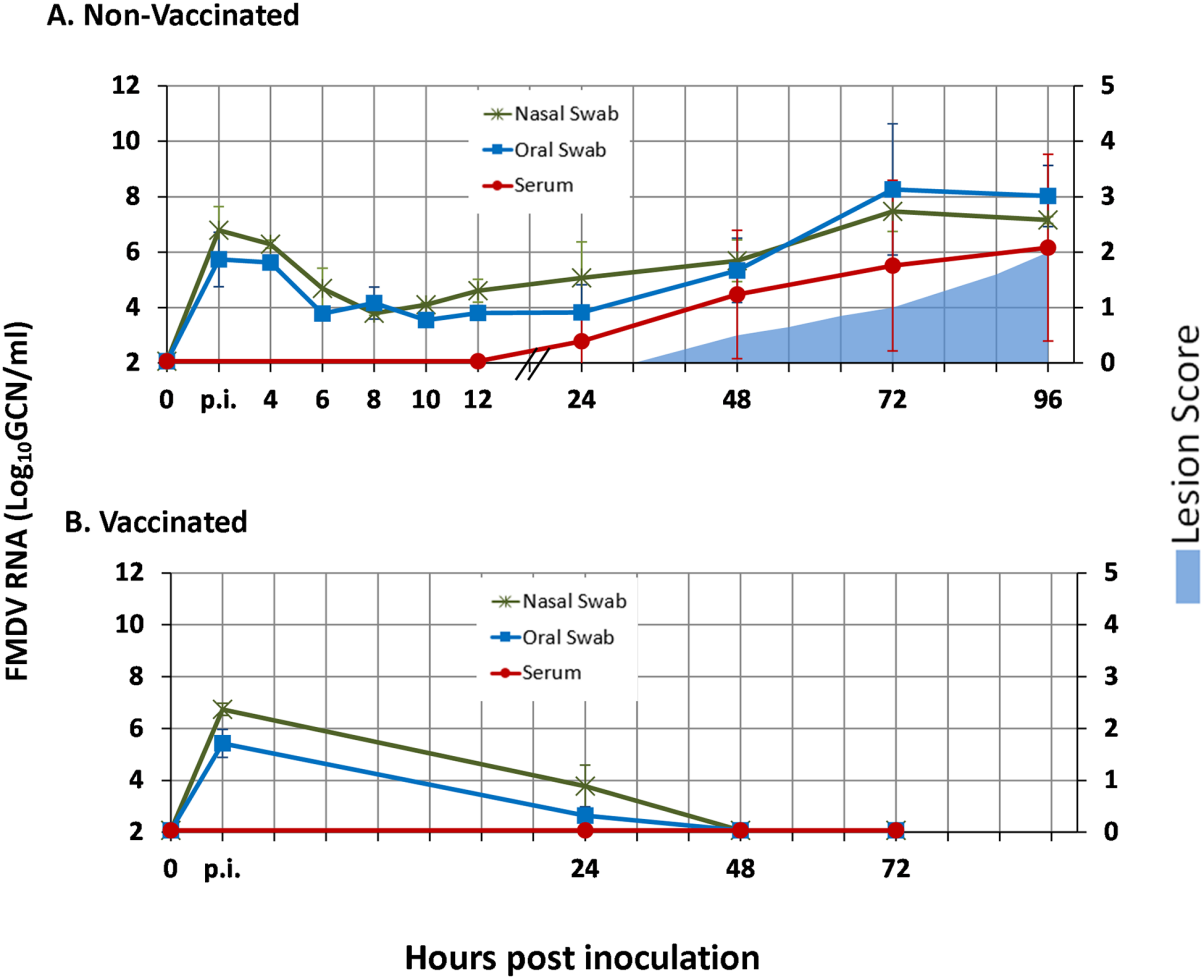
Antemortem infection dynamics in non-vaccinated and vaccinated steers following intra-nasopharyngeal inoculation with FMDV A24 Cruzeiro. A) Non-vaccinated steers, n = 10. B) Vaccinated steers, n = 6. FMDV RNA detection in serum, oral and nasal swabs was performed through qRT-PCR and is presented as log_10_ genome copy numbers (GCN)/ml. Data presented are average values (mean +/- SD) based on samples collected from all cattle included at each time point. “p.i.” (“post-inoculation”) on time scale indicates swab samples harvested directly following inoculation.

Tissue macerates, serum and swab samples were analyzed using a qRT-PCR system, targeting the 3D region of the FMDV genome [28], which encodes the viral polymerase and constitutes a region that is highly conserved across different FMDV serotypes. Forward and reverse primers were adapted from Rasmussen et al [29], with chemistry and cycling conditions as previously described [30]. Cycle threshold (Ct) values were converted to RNA copies per milliliter or milligram using an equation derived from analysis of serial 10-fold dilutions of *in vitro* synthesized FMDV RNA of known concentration. The equations of the curve of RNA copy numbers versus Ct values were further adjusted for average mass of tissue samples and specific dilutions used during processing of samples. The qRT-PCR results reported in Tables 1 and 2 are the higher genome copy number (GCN)/ mg of the 2 samples processed per tissue per animal. Results reported in Fig 1 represent the mean (± SD) log_10_ GCN/ml of all animals sampled at each time point.

### Virus isolation

Aliquots of all individually macerated tissue samples were cleared from debris and potential bacterial contamination by centrifugation through Spin-X filter columns (pore size 0.45μm, Sigma-Aldrich). Tissue macerates and sera were subsequently analyzed for infectious FMDV through virus isolation (VI) on Lois and Frances Bovine Kidney cells transfected with the FMDV receptor, αvβ6 integrin (LFBK αvβ6 cells), a continuous kidney cell line which has been demonstrated to be exquisitely sensitive to FMDV infection [31, 32]. VI was performed following a protocol previously described [3]. Presence of FMDV was further confirmed by qRT-PCR analysis of VI cell culture supernatants from the first cell culture passage.

### Quantification of type I/III interferon bioactivity in nasopharyngeal tissues

An Mx A protein chloramphenicol acetyl transferase (Mx-CAT) reporter gene assay was used to quantitate type I/III interferon (IFN) bioactivity in serum and nasopharyngeal tissue samples as previously described [33, 34]. IFN bioactivity was expressed as IU/ml of serum or IU/g of tissue adjusted for the dilution of tissue in media during processing.

### Laser capture microdissection

Samples of dorsal nasopharynx obtained from non-vaccinated steers euthanized at 12, 24, 48 and 72 hpi and from vaccinated steers euthanized at 24, 48 and 72 hpi, were analyzed using laser capture microdissection (LCM) to determine the micro-anatomic distribution of FMDV RNA during early infection. Ten μm cryosections were cut from two replicate tissue samples of dorsal nasopharynx from each steer. Sections were mounted on metal-framed PEN-membrane slides (LCM0521, Life Technologies), which were kept over a bath of liquid nitrogen until further processing. Slides were dehydrated and stained using the Arcturus Histogene LCM staining kit (KIT0401, Life Technologies) following the manufacturer’s instructions. Four distinct samples representing pre-defined micro-anatomic structures (non-lymphoid epithelium, follicle-associated epithelium, mucosa-associated lymphoid tissue and submucosa; S1 Fig) were dissected from each cryosection (two sections per animal) using an Arcturus XT LCM system. Dissected samples of 150 000 μm^2^ in surface area were captured onto individual CapSure Macro LCM caps (LCM0211, Life Technologies) which were immediately mounted onto micro-tubes containing 50μl of RNA extraction buffer (PicoPure). The LCM procedure was optimized so that the time elapsed from dehydration of tissue sections until dissected samples were submerged in extraction buffer did not exceed 20 minutes. RNA extraction was performed using the PicoPure RNA isolation kit (KIT0202, Life Technologies) with a final elution volume of 17μl.

#### Quantification of FMDV RNA in LCM samples

FMDV RNA contents in distinct micro-anatomic compartments were measured using the qRT-PCR protocol described above, accounting for specific elution and dilution volumes used through the procedure. The weight of the sample input in the qRT-PCR assay was estimated based on sample volume (150 000μm dissected area x 10μm section thickness) and a soft tissue density of 1.0g/cm^3^. The entire procedure, from cryosectioning of tissues to qRT-PCR of dissected samples, was performed on the same day, without freezing of samples between processing steps.

#### Quantification of host cytokine induction in LCM samples

Quantitation of IFN -α, -β, -γ, and -λ mRNA was measured in total RNA extracted from the micro-dissected tissue samples described above. The assay was performed using previously described primers and probes [33] with glyceraldehyde 3-phosphate dehydrogenase (GAPDH) as reference gene. Extracted RNA was diluted 5-fold before qRT-PCR analysis was performed using chemistry and cycling conditions described above. Baseline mRNA expression levels for each cytokine within each distinct tissue compartment were established through similar analysis of four distinct tissue samples from each of three un-infected control steers, generating 12 baseline measurements for each micro-anatomic compartment (4 tissues x 3 animals). Relative expression of each host gene was calculated using the ΔΔCt method [35].

#### Statistical analysis of host cytokine induction in LCM samples

LCM qRT-PCR data was analyzed with a mixed-effects model for each log-transformed dependent variable (DV); IFN-α, -β, -γ, -λ, or FMDV GCN/mg, using a similar approach as described previously [36]. Candidate models were constructed with vaccination status, disease progression category (category I, II or III for non-vaccinated animals and 24, 48, or 72 hpi for vaccinated animals) and micro-anatomical region (Epith = non-lymphoid epithelium; FAE = lymphoid follicle-associated epithelium; LF = mucosa-associated lymphoid tissue; SM = submucosa) as categorical predictors, FMDV RNA load as a covariate (for cytokine DVs), and included possible interactions between them. Animals and tissue replicates were included as random effects. A combined model for all cytokine DVs was constructed analogously.

The best model for each dependent variable was selected based on the second-order Akaike information criterion (AICc) and used for post-hoc analyses. P-values <0.05 were considered significant. All data analysis was performed using the R statistical environment with the reshape2, lme4, AICcmodavg, car, phia, lsmeans and ggplot2 packages.

### Immunomicroscopy

After screening of tissue samples for FMDV RNA using qRT-PCR, detection of antigen in cryosections was performed by immunohistochemistry (IHC) and multichannel immunofluorescence (MIF) as previously described [2, 37]. Slides were examined with a wide-field, epifluorescent microscope, and images were captured with a cooled monochromatic digital camera. Images of individual detection channels were adjusted for contrast and brightness and merged in commercially available software (Adobe Photoshop CS2). Commercially available isotype-control antibodies (Invitrogen, MG100 and MG2B00) were included as negative controls for FMDV detection, with additional negative control tissue sections prepared from corresponding tissues from non-infected cattle. Non-structural FMDV 3D protein was detected using a mouse monoclonal antibody F19-6 [38] and FMDV structural protein VP1 was detected using mouse monoclonal antibody 6HC4 [39]. MIF experiments included labeling of phenotypic cell markers using the following antibodies: poly-clonal rabbit anti-cytokeratin (Life Technologies, 180059), mouse monoclonal anti-bovine CD8 (AbD Serotec, MCA837G), anti-bovine CD3 (clone MM1A, Washington State University, BOV2009), anti-sheep MHC II (AbD Serotec, MCA2228) and anti-bovine CD11c (clone BAQ153A, Washington State University, BOV2026).

## Results

### Antemortem infection dynamics

#### Infection dynamics in non-vaccinated steers

Oral and nasal swabs collected immediately after intra-nasopharyngeal inoculation contained abundant FMDV RNA (5.4 and 6.5 (mean) log_10_ GCN/ml, respectively; Fig 1A and S1 Table). This residual inoculum had declined by 6 hpi in the non-vaccinated steers that were subjected to intensive sample collection after inoculation (Fig 1A and S1 Table). Viral RNA remained at low but consistently detectable levels in oral and nasal swabs before nasal shedding started to gradually and continuously increase at 8–10 hpi, followed by an increase in oral shedding at 24–48 hpi (Fig 1A). Quantities of viral RNA in nasal swabs were slightly higher than in oral swabs during the incubation period, although this trend reversed at the onset of the clinical phase of infection, consistent with the development of vesicular lesions in the oral cavity. Viremia (defined as the combined detection of FMDV RNA and infectious virus in serum) was first detected at 24 hpi (FMDV RNA levels in serum for individual animals are tabulated in Table 1). There was some variation in the timing of onset of viremia and clinical disease within the group (Table 1), with a prolonged pre-viremic phase in a subset of animals (range 24 to 72 hours). However, all animals had consistent detection of viral RNA in oral and nasal swabs, indicating that they were indeed infected by inoculation. Additionally, there was a uniformly marked increase in shedding of viral RNA occurring concurrent with onset of viremia which occurred approximately 24 hours prior to appearance of vesicular lesions.

Characteristic vesicular lesions were present in the oral cavity and on the feet of non-vaccinated steers that were kept alive long enough to develop clinical FMD. The earliest lesions were observed at 48 hpi, although there was some variation in the timing of lesion development within the group. Occurrence of vesicular lesions was often, though not consistently, accompanied by a transient increase in rectal temperature (≥40°C; not shown). Oral lesions consisted of epithelial blanching and vesiculation, primarily on the dental pad and gingival mucosa, and on the dorsal surface of the tongue. Ruptured vesicles formed erosions, which in some instances were the first lesions observed. Foot lesions consisted of vesicles within the inter-digital cleft or on the coronary bands and were often accompanied by lameness.

#### Infection dynamics in vaccinated steers

Similar as in non-vaccinated steers, abundant levels of FMDV RNA were detected in oral and nasal swabs collected from vaccinated steers immediately after inoculation. However, FMDV shedding declined to below detectable levels by 48 hpi in the vaccinated steers (Fig 1B and S1 Table). There was no detection of viral RNA in the blood of any of the vaccinated steers at any time after virus challenge.

None of the vaccinated steers developed any signs of clinical disease. This was also consistent in 22 vaccinated and challenged animals that were kept alive for longer duration.

### Tissue distribution of FMDV

#### FMDV tissue distribution in non-vaccinated animals

Non-vaccinated steers were euthanized at five pre-determined time points after inoculation, regardless of progression of clinical disease (Table 1). As the progression of infection over time was not entirely synchronous across steers, non-vaccinated cattle were grouped by disease progression (clinical disease and viremia) for tabulation and data analyses. This alignment resulted in three distinct disease progression categories: Category I: pre-viremic/pre-clinical (steers no. 1–5), Category II: viremic/pre-clinical (steers no. 6 and 7), and Category III: viremic/clinical (steers no. 8–10).

Category I, pre-viremic/pre-clinical, included a subset of animals euthanized at time points ranging from 12 to 72 hpi (Table 1, steers 1–5). Low quantities of FMDV RNA were detected in serum from two steers within this group (steers 4 and 5; Table 1), but virus isolation was consistently negative from sera of all animals in this category. In pre-viremic animals, the most consistent detection of FMDV RNA and infectious virus was found within the nasopharynx. FMDV RNA was detected in the dorsal nasopharynx (rostral and caudal) and caudal dorsal soft palate from all but one animal within this category (Table 1). Infectious virus (without detectable viral RNA) was detected in the caudo-dorsal nasopharynx from the remaining steer. The nasopharyngeal mucosal samples also contained the highest quantities of viral RNA within this group of animals (up to 6.77 log_10_ GCN/mg). Infectious virus and/or lower quantities of viral RNA (≤ 3.52 log_10_ GCN/mg) were also detected at other sites, including the lingual and palatine tonsils as well as some samples of pulmonary tissue and lymph nodes (Table 1).

Category II, viremic/pre-clinical, consisted of two animals which were euthanized at 24 and 96 hpi, respectively. These steers had not yet developed vesicular lesions, but were confirmed to be viremic based on combined detection of infectious virus and viral RNA in serum (Table 1, steers 6 and 7). Virus distribution (FMDV RNA and infectious virus) in tissues was predominantly localized to the nasopharynx in one of the two steers, with additional virus detection in the tongue and oropharyngeal tonsils in this same animal. Virus detection in tissues from the other category II steer was more disseminated, also including detection of infectious virus and viral RNA in all segments of lungs, with higher quantities of viral RNA found in cranial pulmonary lobes.

Category III, viremic/clinical animals, included three steers euthanized between 48 and 96 hpi, which all had clinical FMD with vesicular lesions in the oral cavity and on one or two feet (Table 1, steers 8–10). Infectious FMDV and viral RNA were widely disseminated in these animals, coincident with high levels of FMDV RNA and infectious virus in the circulation. The highest quantities of viral RNA were found at sites of vesicular lesions in samples harvested from the inter-digital clefts, coronary bands, or tongue. These samples contained FMDV RNA quantities substantially higher than those detected in serum, consistent with viral amplification occurring at these sites (Table 1). FMDV RNA quantities within the nasopharyngeal mucosa remained at levels comparable to those found in earlier phases of disease progression. These levels were close to, or slightly greater than the quantities of virus detected in serum. Viral RNA and infectious virus were consistently detected in the lungs of animals of the viremic/clinical category. FMDV RNA quantities in pulmonary tissues were close to serum levels, though not high enough to provide convincing evidence of viral amplification occurring in the lungs.

#### FMDV detection in tissues from vaccinated animals

Vaccinated animals were euthanized at 24, 48 and 72 hours after virus challenge (Table 2). FMDV RNA was detected in tissues of four out of six steers, with infectious virus isolated from tissues of five out of six steers. Detection of FMDV RNA and infectious virus was largely limited to the nasopharyngeal mucosa, with some additional positive samples detected in oropharyngeal tonsils and mucosa as well as VI-positivity in retropharyngeal and hilar lymph nodes of one steer euthanized at 24 hpi (Table 2). In contrast to non-vaccinated animals, there was no apparent progression in tissue distribution of virus over time within the vaccinated group. However, the quantities of FMDV RNA detectable in nasopharyngeal mucosa from vaccinated steers were comparable to those found in corresponding anatomic sites in non-vaccinated animals, regardless of the time point of euthanasia.

### Micro-anatomic distribution of FMDV RNA in pharyngeal mucosa

FMDV RNA distribution across four distinct micro-anatomic compartments (S1 Fig) was determined in samples of dorsal nasopharynx from eight non-vaccinated and six vaccinated steers, using laser capture microdissection (LCM) combined with qRT-PCR. In non-vaccinated, pre-clinical steers (categories I and II), FMDV RNA was detected only within follicle-associated epithelium overlying lymphoid-rich MALT tissue (Table 3). In viremic/clinical animals (category III), viral RNA was also localized to the submucosal tissue compartment but not within subepithelial lymphoid follicles. Thus, viral RNA was not detected in non-lymphoid epithelium or lymphoid follicles of non-vaccinated animals. In contrast to this, FMDV RNA was detected most consistently within mucosa-associated lymphoid tissue (subepithelial MALT follicles) in nasopharyngeal tissue samples obtained from vaccinated cattle (Table 3). FMDV RNA was detected in microdissected MALT follicles in four out of six cattle of this co-hort (Table 3). Additionally, there was concurrent detection of viral RNA in follicle-associated epithelium and/or non-lymphoid epithelium in two of these animals. Viral RNA was not detected in LCM-generated samples from one non-vaccinated (number 1) and two vaccinated (numbers 12 and 15) steers, which was consistent with negative FMDV qRT-PCR findings in all (macroscopically screened) pharyngeal tissue samples harvested from these animals (Tables 1, 2 and 3).

**Table 3:** Micro-anatomic distribution of FMDV RNA in nasopharyngeal mucosa of non-vaccinated and vaccinated animals determined by laser capture microdissection.

	**Non-Vaccinated steers**							
	*Category I*					*Category II*	*Category III*	
*Time point (hpi)*	***12***	***12***	***24***	***48***	***72***	***24***	***48***	***72***
***Animal ID***	***1***	***2***	***3***	***4***	***5***	***7***	***8***	***9***
***Micro-anatomic region***	*Log*_*10*_ *FMDV GCN/mg tissue* *							
Non-lymphoid Epithelium	-	-	-	-	-	-	-	-
Follicle Associated Epithelium	-	2.55	5.95	5.57	2.87	4.66	-	4.25
Mucosa Associated Lymphoid Tissue	-	-	-	-	-	-	-	-
Submucosa	-	-	-	-	-	-	3.30	4.41
	**Vaccinated Steers**							
*Time point (hpi)*	***24***				***48***		***72***	
***Animal ID***	***11***		***12***		***13***	***14***	***15***	***16***
***Micro-anatomic region***	*Log*_*10*_ *FMDV GCN/mg tissue* *							
Non-lymphoid Epithelium	3.08		-		-	-	-	-
Follicle Associated Epithelium	5.94		-		5.16	-	-	-
Mucosa Associated Lymphoid Tissue	3.86		-		3.22	4.72	-	2.79
Submucosa	-		-		-	-	-	-

* Sample weight calculated on basis of dissected sample volume and tissue density.

### Quantitation of interferon mRNA in micro-dissected samples of nasopharyngeal tissue

**Fig 2:**
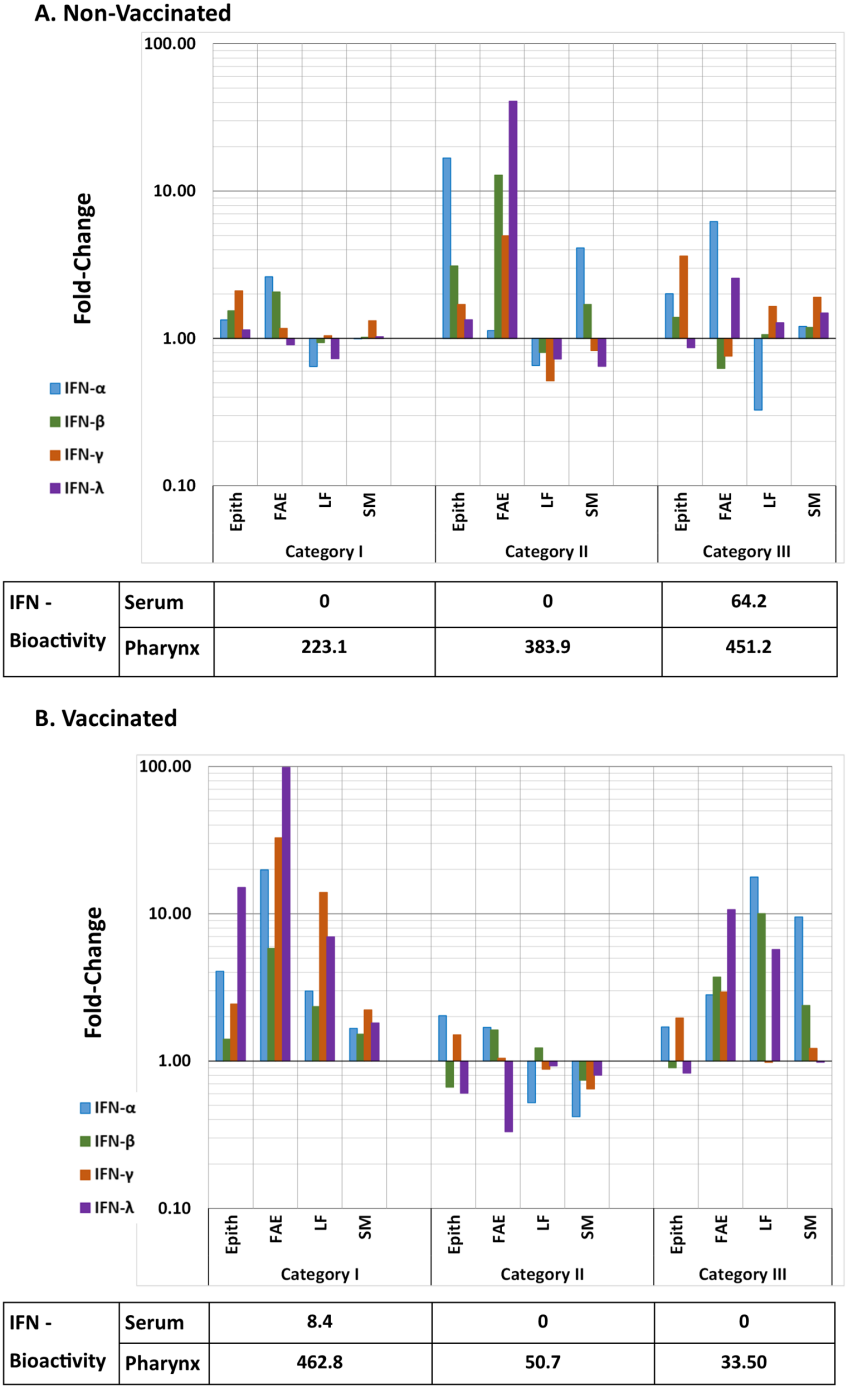
IFN mRNA expression in micro-dissected nasopharyngeal mucosa with systemic and local interferon bioactivity. A) Non-Vaccinated and B) Vaccinated. IFN-α, -β, -γ, and -λ mRNA expression levels were measured by qRT-PCR in micro-dissected tissue compartments of nasopharyngeal mucosa and reported as mean fold-induction. Non-vaccinated steers are grouped by disease progression: Category I = Pre-Viremic/Pre-Clinical, Category II = Viremic/Pre-Clinical, Category III Viremic/Clinical. Vaccinated steers are grouped by time of euthanasia as there was no viremia or clinical disease in these animals (see Table 2). mRNA expression levels are defined as fold-changes normalized to housekeeping gene (GAPDH) and relative to a baseline established from similar analyses of tissues from non-infected cattle. IFN I/III bioactivity measured in nasopharyngeal whole-tissue macerates (IU/g) and serum (IU/ml) collected on the day of euthanasia for each animal category tabulated below X-axes. Epith = non-lymphoid epithelium, FAE = lymphoid follicle associated epithelium, LF = lymphoid follicle, SM = submucosa (see supplement 1 for micro-anatomic definitions).

Relative expression (normalized to host housekeeping gene GAPDH and compared to uninfected animals) of IFN-α, -β, -γ, and -λ mRNA was quantified in total RNA extracted from LCM-derived samples of nasopharyngeal mucosa. At 24 hpi, mRNA levels of all IFNs were significantly induced in all micro-anatomic compartments of vaccinated steers compared to pre-viremic (category I) non-vaccinated steers, with the greatest induction levels in follicle-associated epithelium (Fig 2B). There was no induction of IFN mRNA in nasopharyngeal mucosa prior to detection of viremia in non-vaccinated steers. IFN induction in non-vaccinated animals was observed during the phase of early viremia (category II steers) and was restricted to epithelial compartments (non-lymphoid epithelium and follicle-associated epithelium) of Category II steers (Fig 2A).

**Fig 3:**
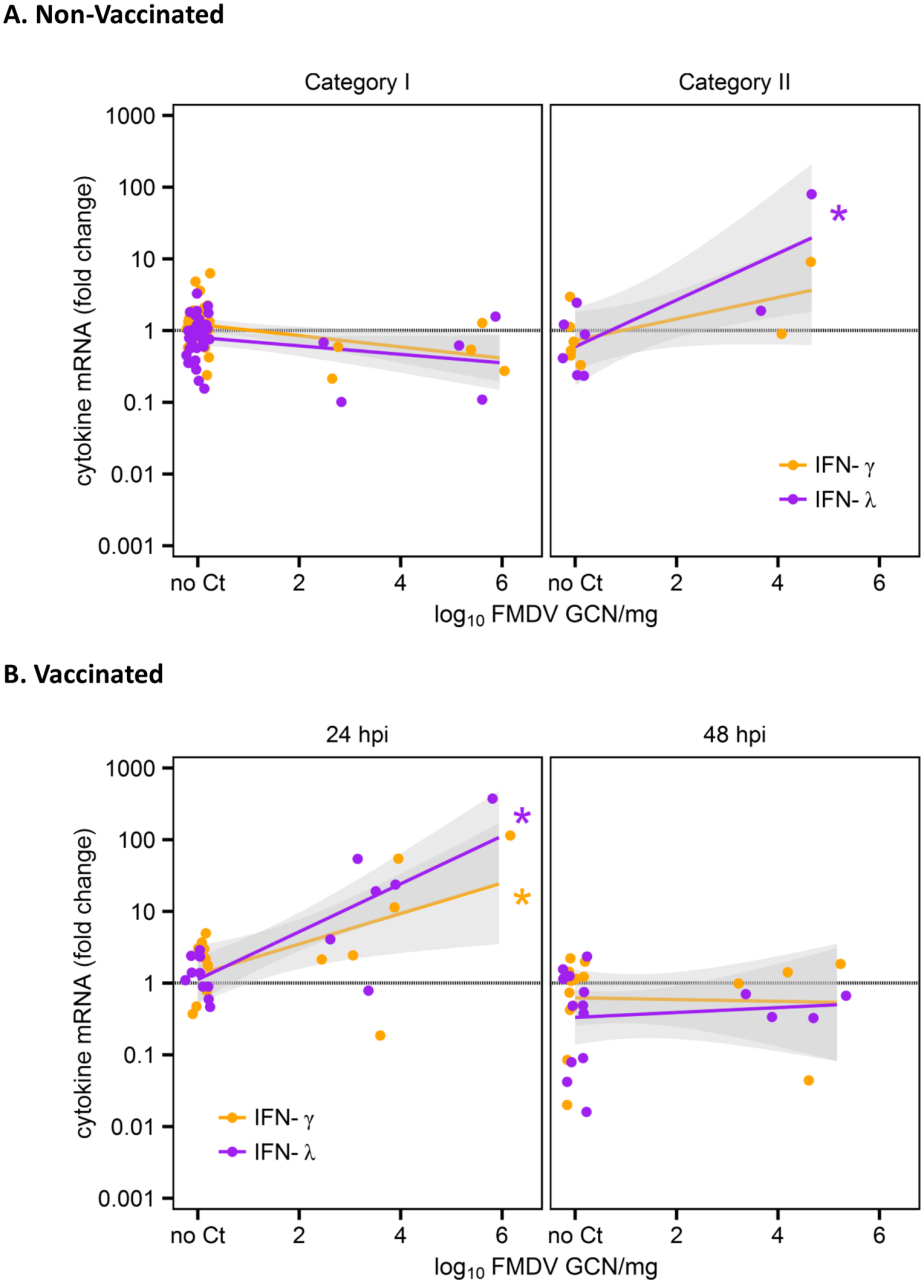
Correlation of FMDV RNA load and IFN-γ, and –λ expression in micro-dissected tissue samples. A) Non-Vaccinated and B) Vaccinated. IFN-γ and -λ mRNA expression levels measured by qRT-PCR in micro-dissected nasopharyngeal mucosa. FMDV RNA load had a significant positive influence on IFN-γ (orange) and IFN-λ (purple) expression in vaccinated animals early after infection (24 hpi), as well as on IFN-λ in non-vaccinated animals that were viremic/ pre-clinical (Category II). Linear regression lines are shown with 95% confidence intervals (gray). Significant correlations are marked with asterisks (*).

The greatest inductions occurred for IFN-γ and IFN-λ, for which the best-fit models included vaccination status, disease progression category (time-point for vaccinated animals) and FMDV RNA load as explanatory variables. FMDV RNA load as a continuous main effect was significant in the IFN-λ model (p < 0.05); IFN-λ and viral RNA load were significantly correlated (Pearson’s ρ = 0.25, p < 0.01). Additionally, there was a highly significant 3-way interaction (category/time-point × vaccination status × FMDV RNA load; p < 0.001): FMDV RNA load had a significant positive influence on IFN-γ and IFN-λ expression in vaccinated animals early after infection (24 hpi; p<0.01 for both), as well as on IFN-λ in non-vaccinated animals that were viremic/pre-clinical (Category II; p<0.001) (Fig 3). For IFN-α gene expression, only the micro-anatomical region remained as a significant explanatory term in the model (p<0.001) and expression levels did not correlate with viral load (ρ = -0.02, p > 0.05). Contrastingly, for IFN-β, FMDV RNA load was the only significant predictor (p < 0.05), and viral load and cytokine expression were significantly correlated (ρ = 0.19, p < 0.05).

There was no consistent modulation of IFN mRNA in the two vaccinated steers euthanized at 48 hpi, whereas there was an overall trend of IFN induction (though not statistically significant) in corresponding samples obtained at 72 hpi (Fig 2B).

### Interferon bioactivity in serum and nasopharyngeal tissue

Type I/III interferon bioactivity was measured in serum collected on the day of euthanasia as well as in whole-tissue macerates of nasopharyneal tissue samples (non micro-dissected samples) by use of an Mx-CAT reporter assay. In non-vaccinated animals, low levels of IFN bioactivity were detected in nasopharyngeal tissues during the pre-viremic phase of infection (Category I) with a subsequent progressive increase coincident with occurrence of viremia and clinical disease (Fig 2A). In these animals, IFN activity in serum was only detectable in animals euthanized during viremia and clinical infection (Category III; Fig 2A). Contrastingly, in vaccinated animals, IFN bioactivity in nasopharyngeal tissues at 24 hpi was higher than in pre-clincial, non-vaccinated animals (Categories I and II). However, in contrast to the temporal progression in non-vaccinated animals, IFN bioactivity in serum and nasopharyngeal tissue of vaccinated cattle returned to below or near-baseline levels at subsequent time points (48–72 hpi) (Fig 2B).

### Microscopic distribution of FMDV in the context of phenotypic characterization of host cells

**Fig 4:**
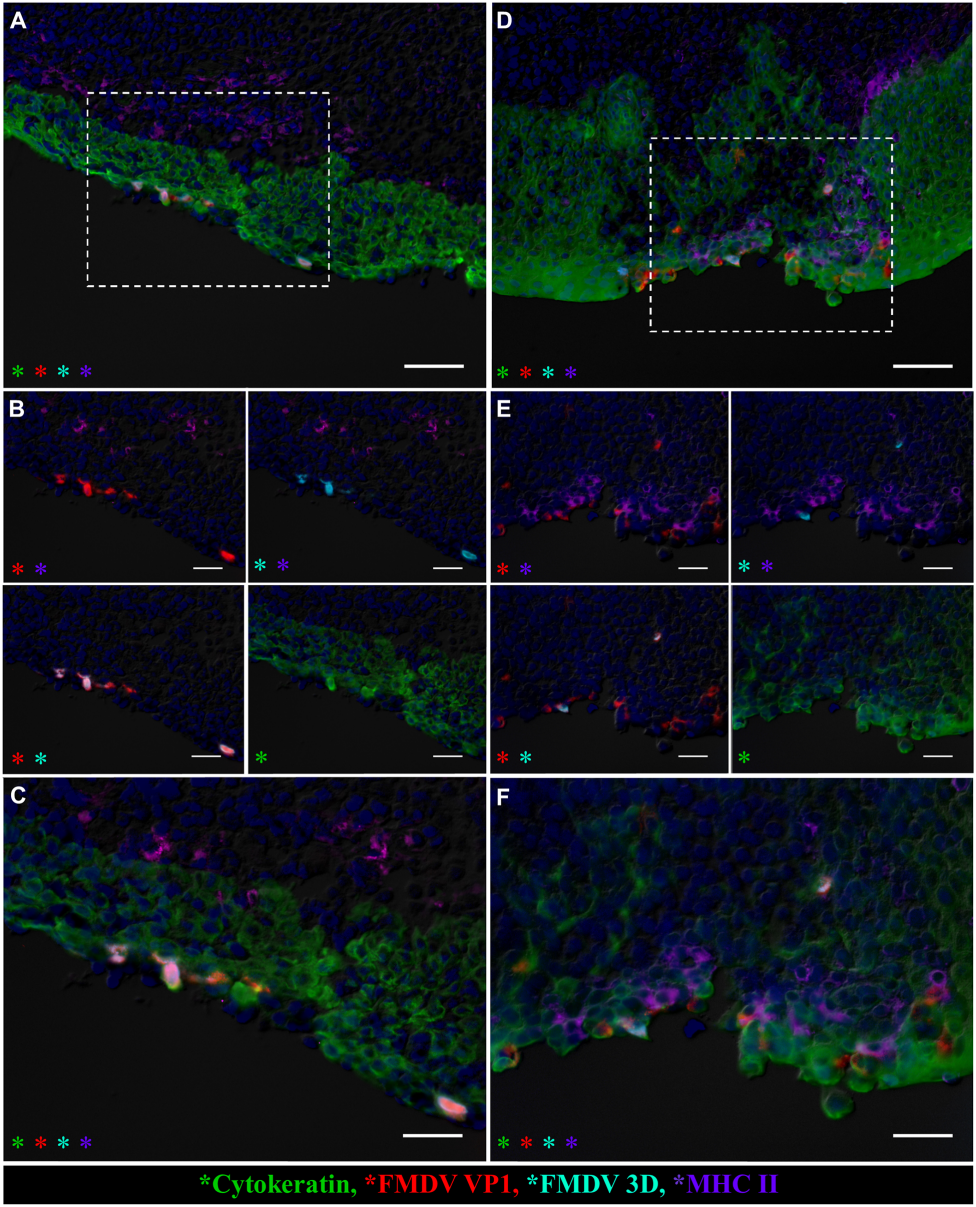
Primary infection of nasopharyngeal mucosal epithelial cells is similar in non-vaccinated and vaccinated cattle. Microscopic distribution of FMDV structural (VP1) and non-structural (3D) protein in dorsal nasopharyngeal mucosa of non-vaccinated (A-C) and vaccinated (D-F) cattle at 24 hpi. Multichannel immunofluorescent technique. **A)** Nasopharyngeal mucosa of non-vaccinated steer at 24 hpi (animal number 3). FMDV VP1 (red) and 3D (aqua) proteins co-localize with cytokeratin (green) in foci of primary FMDV infection in superficial layer of follicle-associated epithelium. Scarce MHC II+ cells (purple) are present in the subepithelium. 20x magnification, scale bar 50μm. **B-C)** 40x magnification of region identified in (A). **B)** Select channel combinations demonstrating co-localization of FMDV VP1(red) and FMDV 3D (aqua) with cytokeratin (green), but not with MHC II (purple) **C)** Merge of all images shown in (B), scale bar 25μm. **D)** Nasopharyngeal mucosa of vaccinated steer at 24 hpi (animal number 11). FMDV VP1 (red) and 3D (aqua) proteins co-localize with cytokeratin (green) within follicle associated epithelium. Intra-epithelial MHC II+ cells (purple) are in close proximity to virus-infected epithelial cells. 20x magnification, scale bar 50μm. **E-F)** 40x magnification of region identified in (D). **E)** Select channel combinations demonstrating FMDV VP1 (red) and 3D (aqua) colocalization with cytokeratin (green)-expressing cells and with MHC II- expressing cells (purple). **E)** Merge of images shown in (F), scale bar 25μm.

**Fig 5:**
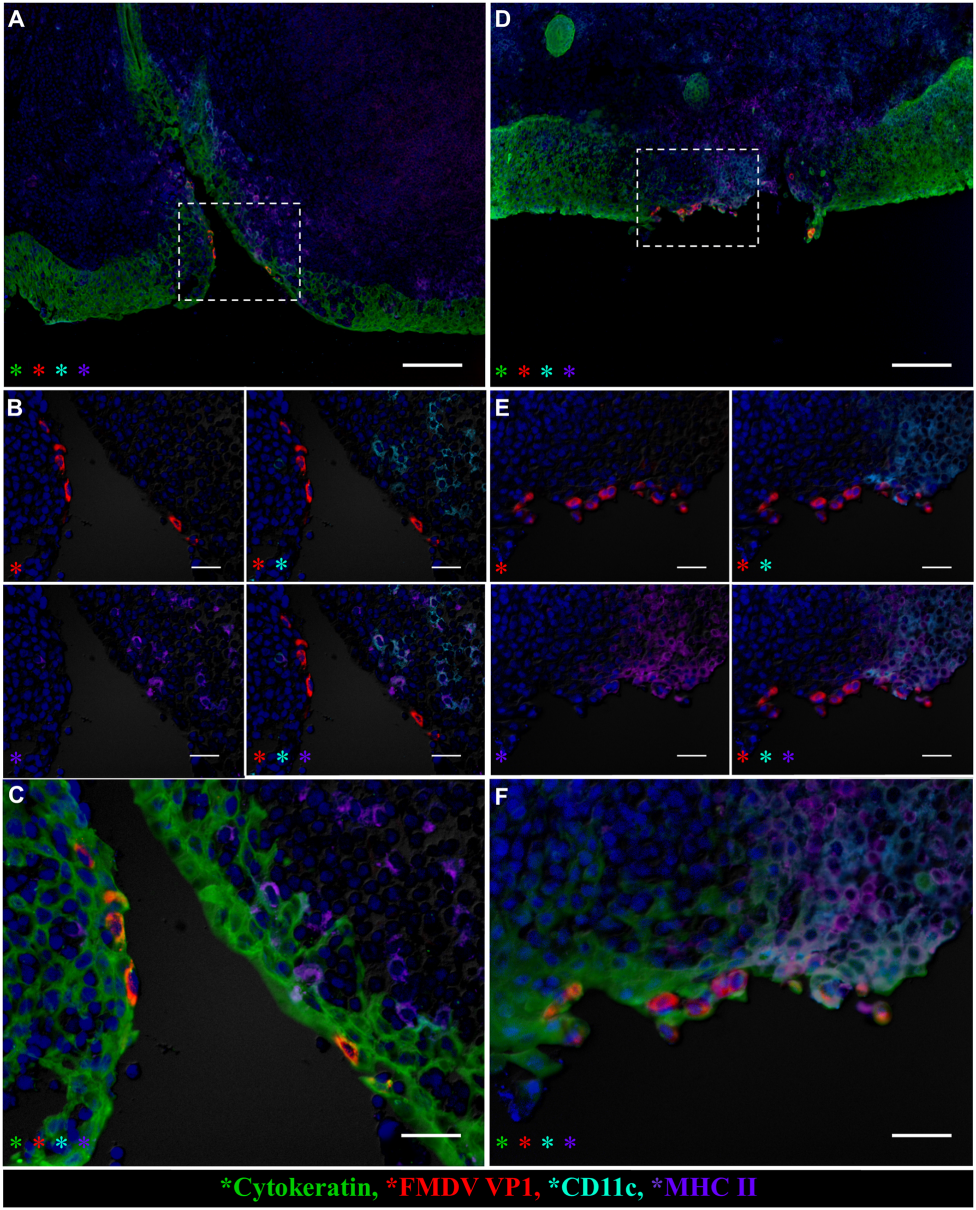
Differential host-virus interactions in nasopharyngeal mucosa of non-vaccinated (A-C) versus vaccinated (D-F) cattle. Multichannel immunofluorescent technique. **A)**. FMDV VP1 (red) protein within cytokeratin^+^ cells (green) in epithelial crypt of the nasopharyngeal mucosa of non-vaccinated steer at 24 hpi (animal number 3). Host cells expressing MHC II (purple) and/or CD11c (aqua) are present in the subepithelial compartment and interspersed within epithelium, but without co-localizing with viral protein. 10x magnification, scale bar 100μm. **B-C)** 40x magnification of region identified in (A), scale bars 25μm. **B)** Select channel views demonstrate lack of co-localization of FMDV VP1 (red) with CD11c (aqua)/ MHC II (purple)-double positive cells. **C)** Merge of all images shown in (B), scale bar 25 μm. **D)** FMDV VP1 (red) protein co-localizes with cytokeratin (green) in an epithelial erosion in dome region of follicle-associated epithelium of nasopharyngeal mucosa of vaccinated steer at 24 hpi (animal number 11). Regionally extensive infiltration by MHC II (purple) and/or CD11c (aqua)-expressing cells co-localizing and interspersing with viral antigen.10x magnification, scale bar 100μm. **E-F)** 40x magnification of region identified in (D), scale bar 25μm. **E)** Select channels demonstrating co-localization of FMDV VP1 (red) with CD11c (aqua), MHC II (purple). **F)** Merge of images shown in (E), scale bar 25 μm.

**Fig 6:**
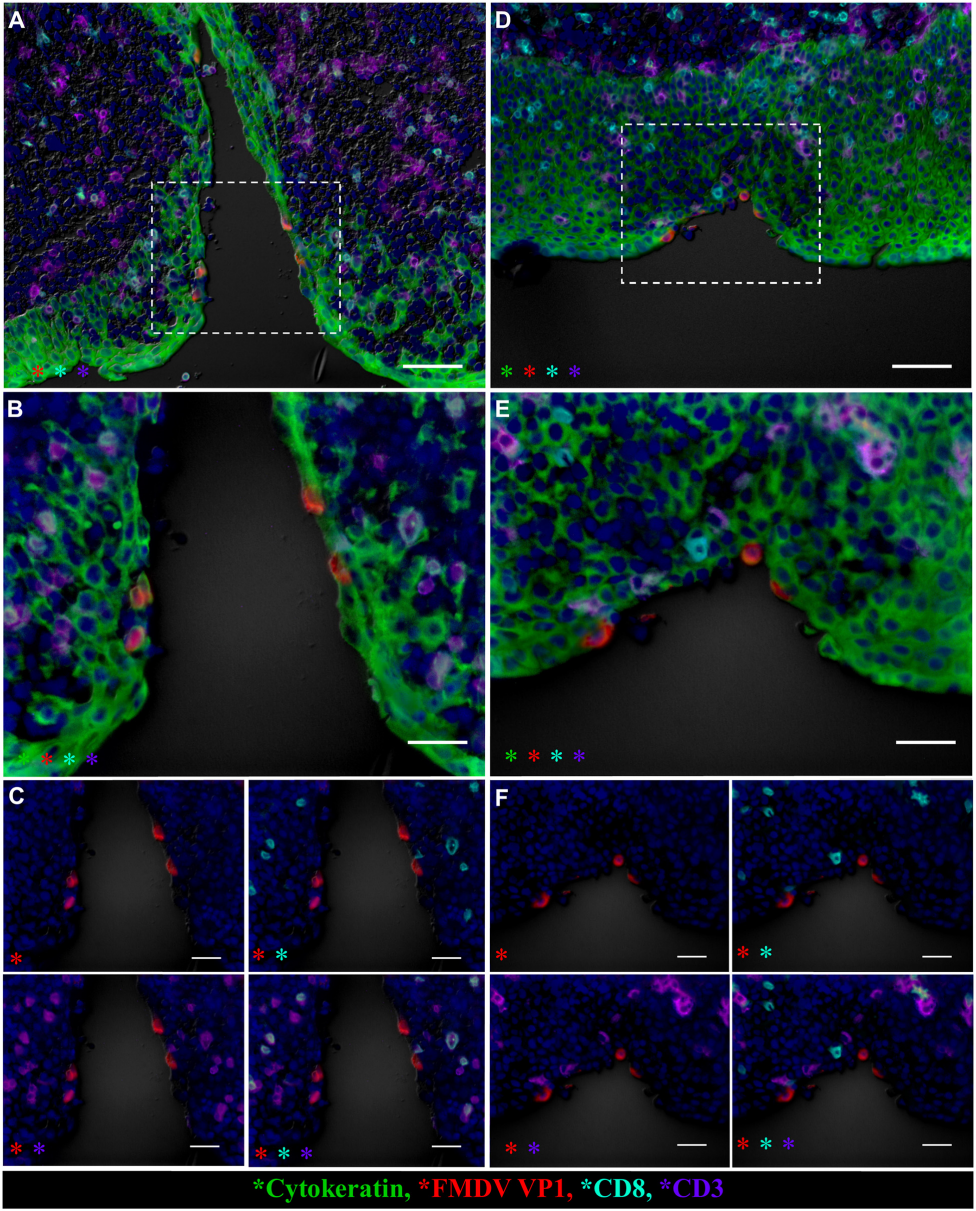
Nasopharyngeal primary infection sites of vaccinated cattle are infiltrated by CD8^+^/CD3^-^ (presumptive NK) cells at 24 hpi. Multichannel immunofluorescent technique. **A)**. FMDV VP1 (red) protein within cytokeratin^+^ cells (green) in epithelial crypt of the nasopharyngeal mucosa of non-vaccinated steer at 24 hpi (animal number 3). CD8^+^ (aqua)/ CD3^+^ (purple) double-positive CTLs are present in the subepithelial compartment amongst larger populations of CD8^-^/CD3^+^ T-lymphocytes. 20x magnification, scale bar 50μm. **B)** 40x magnification of region identified in (A), demonstrating consistent co-localization of CD8 (aqua) with CD3 (purple). Scale bars 25μm. **B)** Select channels of image shown in (B). CD3^+^ cells (purple) include single-positive (T-lymphocytes) or CD8^+^/CD3^+^ double-positive (CTLs). CD8 (aqua) is exclusively detected in combination with CD3 (purple). Scale bars 25 μm. **D)** FMDV VP1 (red) protein co-localize with cytokeratin (green) in a focal surface erosion within follicle-associated epithelium of nasopharyngeal mucosa of vaccinated steer at 24 hpi (animal number 11). A distinct population of cells defined as presumptive NK-cells based on a CD8^+^ (aqua)/CD3^-^ (purple) phenotype is present in submucosal and epithelial compartments surrounding the focus of infection. A smaller population of CTLs (CD8^+^/CD3^+^) is present amongst abundant non-CTL T-lymphocytes (CD8^-^/CD3^+^). 20x magnification, scale bar 50μm. **E)** Higher magnification of region identified in showing CD8^+^/CD3^-^ cells (aqua) representing presumptive NK cells in close proximity of FMDV infected focus. 40x magnification, scale bars 25 μm **F)** Individual channels of image shown in (E) demonstrating inconsistent co-localization of CD8 (aqua) and CD3 (purple). Scale bar 25 μm.

FMDV structural and non-structural proteins were detected within follicle-associated epithelium of the dorsal nasopharynx at 24 hpi in vaccinated steers as well as in pre-viremic, non-vaccinated (Category I) steers (Fig 4). At this early time point, in both groups of animals, viral antigen was present within cytokeratin-positive epithelial cells directly overlying MALT follicles (Figs 4 and 5). FMDV antigen was localized to single cells within surface or crypt epithelium, or as clusters of infected cells associated with small erosions of the epithelial surface (Figs 4 and 5). At the same time point, host cells expressing MHC II and/or CD11c (presumptive antigen-presenting and/or dendritic cells) were detected in close proximity to FMDV-infected epithelial cells in both vaccinated and non-vaccinated animals. However, there was a greater abundance of these cells directly surrounding clusters of FMDV-infected cells in vaccinated animals (Fig 5E and 5F compared to Fig 5B and 5C). Additionally, at 24 hpi, there was co-localization of FMDV structural protein with MHCII+/CD11c+ cells only in the dorsal nasopharynx from a vaccinated steer (Fig 5D–5F). These cells were within the superficial layer of epithelium, interspersed with cytokeratin-positive epithelial cells. Similar co-localization of FMDV antigen with DC markers was never observed in pre-viremic or early viremic non-vaccinated steers (Category I-II). Furthermore, in the vaccinated steers euthanized at 24 hpi, there were abundant CD8^+^/CD3^-^ (presumptive NK) cells in association with foci of primary FMDV infection (Fig 6D–6F). Contrastingly, CD8^+^ cells observed at the corresponding time point in non-vaccinated steers were predominantly CD8^+^/CD3^+^, indicating cytotoxic T-lymphocytes, rather than NK cells (Fig 6A–6C).

**Fig 7:**
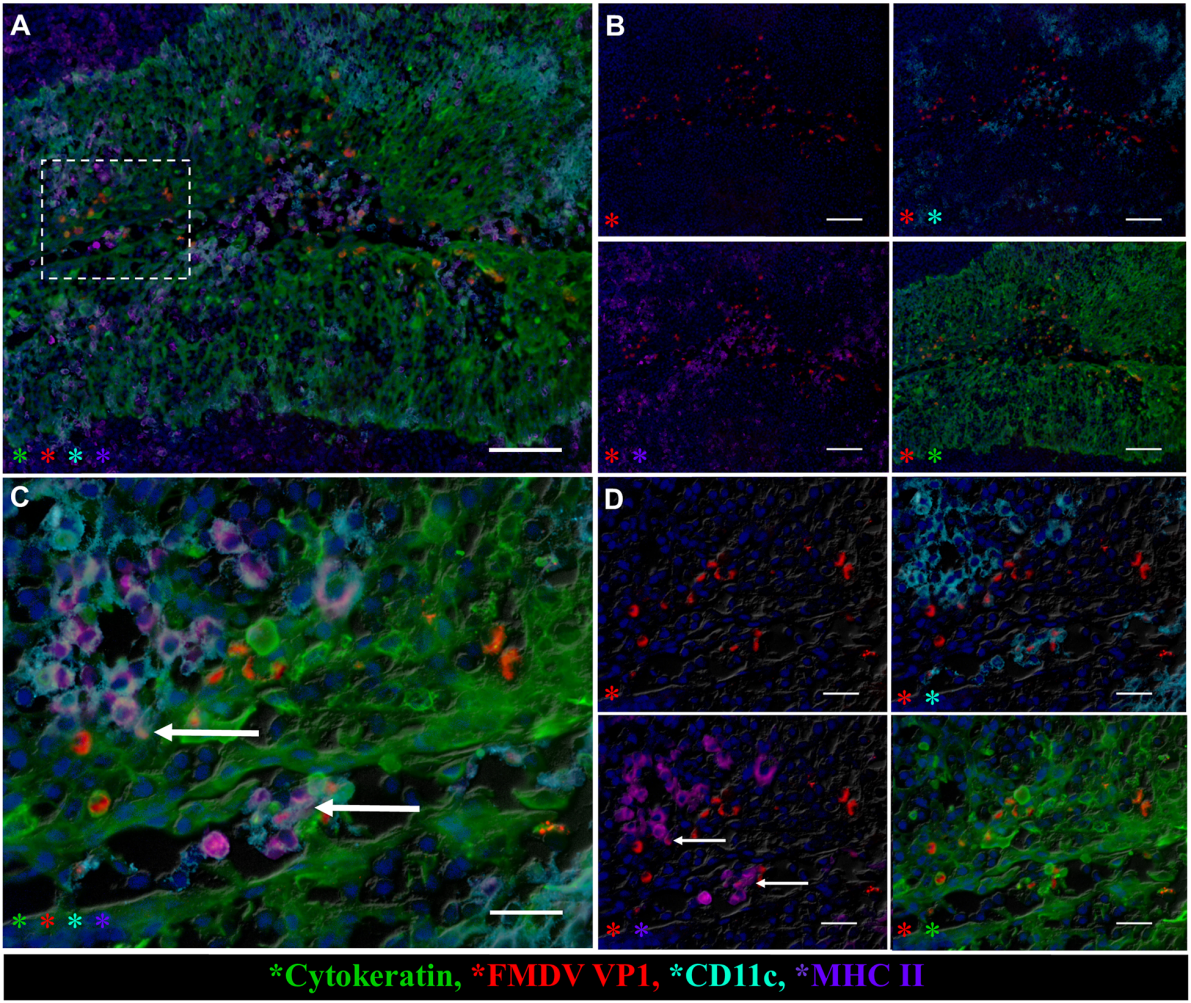
FMDV co-localizes with cytokeratin and MHCII/CD11c-expressing cells in palatine tonsil of non-vaccinated steer at 72hpi. **A)** FMDV VP1 (red) predominantly in cytokeratin-positive cells of epithelial crypt of palatine tonsil of non-vaccinated steer (animal number 9) during viremia and clinical FMD at 72 hpi. Abundant cells expressing MHC II (purple) and/or CD11c (aqua) interspersed with epithelial cells. 10x magnification, scale bar 100 μm. **B)** Select channels of image in (A). FMDV VP1 (red), cytokeratin (green), CD11c (aqua), MHC II (purple) demonstrate FMDV antigen is exclusively within epithelial compartment. **C)** High magnification of region identified in (A). Co-localization with FMDV VP1within subset of MHC II (purple) and/or CD11c (aqua) expressing cells within crypt epithelium (arrows in C & **D)**. 40x magnification, scale bar 25 μm D) Individual channels of image in (B). FMDV VP1 (red), cytokeratin (green), CD11c (aqua), MHC II (purple).

During viremia and clinical infection in non-vaccinated steers, larger foci containing greater quantities of FMDV VP1-positive cells were found within crypt epithelium of the palatine tonsils (Fig 7). FMDV antigen was primarily localized to intact or sloughed cytokeratin-positive epithelial cells in rarefied microvesicular foci. At this advanced stage of disease progression, there was co-localization of FMDV VP1 with CD11c^+^/MHC II^+^ cells (Fig 7A–7D), similar to findings in vaccinated animals at earlier time points.

FMDV antigen was not localized to subepithelial regions by immuno-microscopy in any animals within this study. Numerous additional tissues in which FMDV genome and infectious virus were detected by qRT-PCR and VI were screened microscopically. With the exception of tissues with macroscopic vesicular lesions (including tongue epithelium and coronary band skin), FMDV antigens were exclusively localized to epithelial cells of the nasopharyngeal mucosa and oropharyngeal tonsils.

## Discussion

Multiple works have reported subclinical FMDV infection in vaccinated cattle following virus exposure by documenting shedding of infectious virus in oral, nasal and/or oropharyngeal secretions of virus-challenged cattle [5, 16, 17, 19, 20]. However, there are no previous reports characterizing the specific events of subclinical FMDV infection in vaccinated animals. The objective of the work presented herein was to elucidate and contrast mechanisms of primary FMDV infection in vaccinated and non-vaccinated cattle exposed to FMDV through simulated-natural inoculation. Immunization with a recently licensed recombinant FMDV vaccine 14 days prior to virus exposure conferred complete protection against clinical FMD. This was confirmed by absence of clinical FMD and viremia in 22 additional steers that were included in the studies and kept alive for longer durations (range 15–35 dpi).

There was a marked reduction in shedding of FMDV RNA in vaccinated compared to non-vaccinated steers after virus challenge. Low quantities of viral RNA were detected in oral and nasal secretions of vaccinated animals up to 48 hpi, which is suggestive of viral replication within the upper gastrointestinal or respiratory tracts. However, none of the vaccinated steers developed detectable viremia, and there was no evidence of viral replication at sites distant to the pharynx. In contrast to this, viremia, vesicular lesions and shedding of abundant quantities of viral RNA and infectious virus were present in all non-vaccinated animals that were kept alive long enough to develop clinical FMD.

Subsequent to intra-nasopharyngeal inoculation, primary FMDV infection (determined by concurrent detection of FMDV RNA, infectious virus, and viral antigens) was localized to epithelial cells of the nasopharyngeal mucosa of non-vaccinated cattle. This is consistent with previous works [2–4], which have reported similar findings after exposure to virus through aerosol, contact, or intranasal spray. The current study also demonstrated similar distribution of infectious FMDV in the pharyngeal mucosa of the majority of vaccinated animals that were euthanized at 24–72 hours after virus challenge. The tissue distribution of FMDV genome, infectious virus, and viral antigen in vaccinated steers closely resembled the pre-viremic phase of infection in non-vaccinated steers. Although this anatomic distribution of virus was consistent through the time course of investigation in vaccinated animals, non-vaccinated animals differed by manifesting a marked dissemination of virus in all tissues analyzed concurrent with development of viremia and clinical disease, as has been described previously for other experimental systems [2, 4, 40].

Previous works from our laboratory have demonstrated that in cattle exposed to FMDV via aerosol inoculation, primary infection in the nasopharynx is followed by extensive viral amplification in the lungs, coincident with establishment of viremia and generalized disease [2]. In the current study, localization of primary infection to the nasopharynx was indistinguishable from the previously described aerosol model [2]. Furthermore, INP inoculation led to low levels of FMDV RNA and infectious virus in lungs during the pre-viremic phase of infection in 3 out of 5 non-vaccinated steers, suggesting permissiveness to FMDV replication in pulmonary tissues. Additionally, one early viremic (category II) steer had a transitional distribution of FMDV indicated by detection in all pulmonary tissues prior to detection of any clinical signs of FMD. However, in contrast to the aerosol model, viral quantities in pulmonary tissues never reached levels substantially higher than that detected in blood. Thus, in the current study, there were no indications of substantial virus amplification occurring in the lower respiratory tract.

The contrasting findings between the aerosol and INP models suggest distinct mechanisms of progression of infection dependent on the route of virus exposure. The newly optimized system of intra-nasopharyngeal deposition of virus (INP inoculation) utilized in the current study was developed based on detailed knowledge of FMDV pathogenesis derived from studies using aerosol exposure [2]. The aerosol and INP inoculation systems are both effective simulated-natural inoculation systems for studies of FMDV pathogenesis in cattle. The combined descriptions of these systems indicate that subtle variations in mechanisms of disease progression are dependent upon the experimental system used. However, these differences may reflect variations of FMDV pathogenesis that also occur subsequent to natural FMDV exposure.

Despite similarities in macro-anatomic detection of FMDV RNA and infectious virus in tissues of vaccinated and non-vaccinated steers during early (pre-viremic or subclinical) infection, there were clear differences in micro-anatomic localization of viral RNA as determined by laser capture microdissection. Detection of FMDV RNA within follicle-associated (lymphoid) epithelium of the dorsal nasopharyngeal mucosa was more common in non-vaccinated steers compared to those that were vaccinated. Additionally, exclusively in vaccinated animals, FMDV RNA was detectable in micro-dissected samples obtained from lymphoid (MALT) follicles directly subjacent to the epithelial surface at early stages of infection. This suggests that distinct FMDV pathogenesis events in vaccinated animals differ from pathways in non-vaccinated animals. Viral RNA was detected in the submucosal compartment of non-vaccinated steers during viremia and clinical infection, whilst there was no detection of virus in deeper layers of tissue in vaccinated steers. This finding is consistent with detection of FMDV in the intravascular spaces in viremic animals.

Primary viral replication in follicle-associated epithelium of the dorsal nasopharynx was confirmed by detection of viral antigen by immunomicroscopy. FMDV structural (VP1) and non-structural (3D) proteins were detected within cytokeratin-positive epithelial cells of this specific anatomic region in both vaccinated and non-vaccinated steers at 24 hpi. In both categories of animals, FMDV antigen localized either to scarce individual cells within characteristic segments of crypt epithelium, or as clusters of infected cells forming modest erosions of the surface epithelium overlying MALT follicles. Similar evidence of microscopic epithelial lesions at sites of primary FMDV infection has previously been documented in both cattle and pigs [2, 41].

In the current study, there were several differences in the local host response at the site of primary infection depending on vaccination status. Relative quantities of IFN mRNA in micro-anatomic tissue compartments obtained through LCM indicated significantly greater induction of antiviral activity at the primary infection site of vaccinated steers; this was further corroborated by detection of high IFN bioactivity in these tissues. However, this prompt antiviral induction in vaccinated cattle waned rapidly, decreasing to near-baseline levels over successive time points. There was no detection of a systemic IFN response at any time in the vaccinated animals included herein or within the cohort of vaccinated animals that survived to 35 dpi (data not shown).

Non-vaccinated cattle had distinct patterns of IFN induction, suggestive of different mechanisms between naïve and vaccine-primed innate responses to infection. In this cohort, interferon bioactivity in the nasopharynx increased with clinical and virological progression from Category I to III. Induction of IFN mRNA in the non-vaccinated animals was greatest in Category II (viremic, pre-clinical) despite higher bioactivity in Category III (viremic, clinical). The Category III, non-vaccinated animals were the only group in the study in which there was detection of substantial systemic IFN. This is consistent with previous works which have confirmed a strong correlation between the occurrence of viremia and induction of a systemic IFN response [33, 42, 43], as well as the coincidence of IFN induction in tissues with active viral replication [33].

The findings of the current study demonstrate IFN bioactivity in nasopharyngeal tissue samples of both vaccinated and non-vaccinated cattle during early infection, prior to occurrence of viremia or macroscopic lesions. However, the combined output of IFN mRNA transcriptome analysis and quantification of IFN bioactivity suggest that induction of bioactive IFN occurs by different mechanisms in vaccinated versus non-vaccinated animals. Yet, for both cohorts of animals, the relative induction of INF-γ and -λ correlated positively with viral loads within distinct micro-anatomic compartments although this correlation was not statistically significant for IFN-γ induction in non-vaccinated steers. Overall, these data indicate that complex mechanisms and differential pathways of anti-viral activation may be involved in the early response to FMDV infection in vaccinated and non-vaccinated cattle.

Characterization of cellular phenotypes at sites of primary infection provided further evidence that vaccination induced a distinct, rapid-onset innate immune response subsequent to virus challenge. Specifically, in vaccinated animals there were greater quantities of cells consistent with antigen-presenting cells (APCs) and NK cells at sites of primary infection at early stages of infection. Substantial numbers of CD8^+^/CD3^-^ cells, representing presumptive NK cells [44], were observed in close association with foci of FMDV-infected epithelial cells in the nasopharyngeal mucosa of vaccinated steers euthanized at 24 hpi. Contrastingly, CD8^+^ cells observed at a similar anatomic localization in non-vaccinated steers euthanized at the corresponding time point were consistently CD8^+^/CD3^+^ double-positive, suggesting these cells were either cytotoxic T-lymphocytes (CTLs) or CD8^+^ γ/δ- T-cells. NK cells are part of the innate immune response and cannot be directly linked to a specific vaccine-induced anti-FMDV response. However, previous works have demonstrated that NK-cell activation may occur as part of an adjuvant-induced (antigen-independent) systemic vaccine response [45]. Previous investigations have demonstrated that FMDV infection of non-vaccinated cattle induced a systemic NK response that was not detectable after vaccination with an inactivated vaccine [46]. However, this previous work was based on investigation of PBMCs from vaccinated, non-challenged, cattle that had been inoculated using a different vaccine.

Antigen-presenting cells expressing MHC II and/or CD11c were present at sites of primary infection of vaccinated and non-vaccinated steers. However, vaccinated animals had a more abundant population of MHC II^+^/CD11c^+^ cells (presumptive APCs) within or closely surrounding foci of FMDV infection. The CD11c^+^/MHC II^+^ phenotype has specifically been associated with bovine conventional dendritic cells (DCs) in peripheral blood [47]. These cells are specialized antigen-presenting cells that bridge the innate and adaptive immune response by presenting processed antigen to lymphocytes. Previous studies have demonstrated that activation of DCs by FMDV is substantially enhanced in the presence of FMDV-specific antibodies [48, 49]. Consistent with this concept, co-localization of FMDV VP1 with presumptive APCs of a CD11c+/MHC II+ phenotype was observed in the nasopharyngeal mucosa of vaccinated steers as early as 24 hpi whereas the non-vaccinated cattle had relatively few MHC II^+^/CD11c^+^ cells within foci of primary FMDV infection. The earliest observation of co-localization of FMDV to presumptive APCs in a non-vaccinated steer occurred at 72 hpi, in crypt epithelium of the palatine tonsil. This finding is consistent with previous works which have demonstrated interaction of FMDV and DCs during advanced stages of disease progression [2].

Increased affinity conferred by opsonization of viral particles by bound immunoglobulin may account for the observed variation in timing and magnitude of the cellular host-virus interactions between vaccinated and non-vaccinated animals [48]. Specifically, uptake of virus by dendritic cells of vaccinated animals may have been facilitated by more abundant anti-FMDV immunoglobulin present due to vaccination [47, 50]. These same events also may have triggered induction of pathways leading to the increased transcription of IFN genes [48] that was demonstrated in tissues from these same animals.

Additionally, facilitated uptake of virus by antigen-presenting cells could enable the prompt localization of FMDV genome to MALT follicles, which was demonstrated in samples obtained by laser capture microdissection in vaccinated, but not in non-vaccinated steers. In a previous publication, it was concluded that serotype O FMDV could be detected by LCM and immunomicroscopy in germinal centers of submandibular lymph nodes as early as 48 hours after inoculation of naïve cattle [51]. In the current study, there was no detection of viral genome in lymph nodes draining the oral cavity and pharynx prior to development of viremia (which was present in non-vaccinated steers only). However, the early localization of FMDV RNA to MALT follicles of vaccinated steers suggests an active translocation of viral particles mediated by host mechanisms.

In conclusion, the work presented herein provides the first detailed characterization of FMDV infection in tissues of the upper respiratory tract of vaccinated cattle. Immunization of cattle using a newly developed recombinant FMDV vaccine conferred complete protection against clinical FMD, but did not prevent subclinical infection within the nasopharynx. Furthermore, it was confirmed that shedding of virus was substantially reduced in vaccinated animals. Similar to previous works, vaccination did not prevent subclinical infection of the upper respiratory tract following virus challenge. The localization of the critical events of primary infection of vaccinates was demonstrated to be anatomically indistinguishable from that which occurred in naïve animals. However, primary infection in vaccinates was associated with enhanced local host responses including increased presence of antigen-presenting cells, NK cells, and induction of interferon. It is likely that these events are critical in protecting animals from dissemination of FMDV. Further investigation and characterization of the virus-host interactions at primary infection sites in vaccinates is likely to continue to provide insights that may contribute towards enhanced countermeasures against FMDV.

## Supporting Information

S1 Fig Histologic specimen of bovine dorsal nasopharynx identifying four specific micro-anatomic regions isolated by laser capture microdissection. (PDF)

S1 Table FMDV RNA quantities in serum, nasal- and oral swabs from individual animals included in the study. (PDF)
