# Supplementary material for: Pathogenesis of Primary Foot-and-Mouth Disease Virus Infection in the Nasopharynx of Vaccinated and Non-Vaccinated Cattle
Source: PLoS One. 2015 Nov 23;10(11):e0143666. doi: 10.1371/journal.pone.0143666 (PMC4658095; doi:10.1371/journal.pone.0143666)
Supplement: S1 Fig — (PDF) [file pone.0143666.s001.pdf]

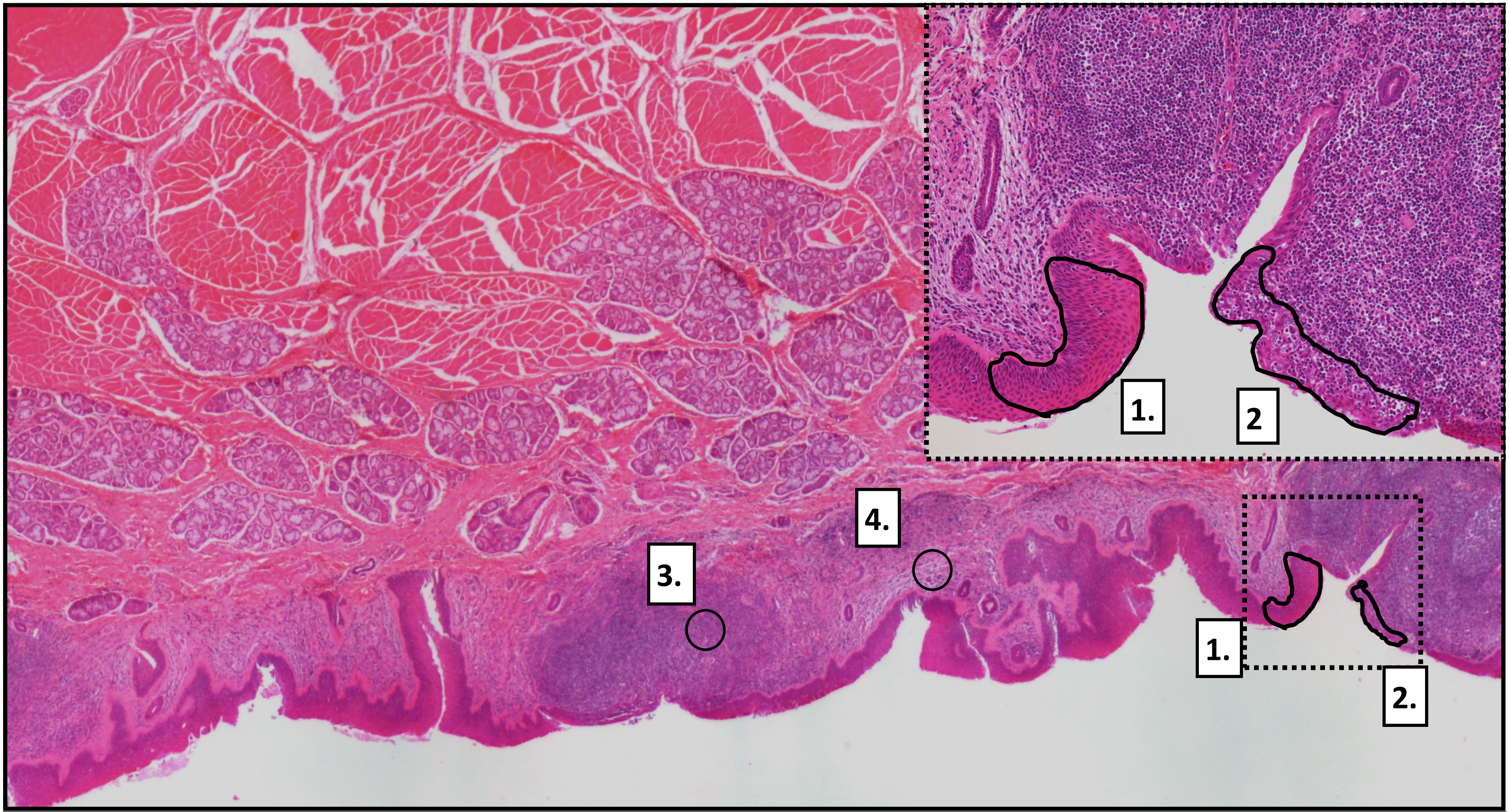

S1 Fig: Histologic specimen of bovine dorsal nasopharyngeal mucosa identifying four distinct micro-anatomic regions isolated by laser capture microdissection. **(1): Non-lymphoid epithelium**, **(2): Follicle associated epithelium**, **(3): Sub-epithelial MALT follicles, (4): Submucosa**. 10x magnification, Hematoxylin and Eosin stain. Inset: 20x magnification of area within hatched square.
