# Supplementary material for: Pathogenesis of Primary Foot-and-Mouth Disease Virus Infection in the Nasopharynx of Vaccinated and Non-Vaccinated Cattle
Source: PLoS One. 2015 Nov 23;10(11):e0143666. doi: 10.1371/journal.pone.0143666 (PMC4658095; doi:10.1371/journal.pone.0143666)
Supplement: S1 Table — (PDF) [file pone.0143666.s002.pdf]

S1 Table

FMDV RNA quantities (log_{10} genome copy number [GCN]/ml) in nasal- and oral swabs and serum from non-vaccinated and vaccinated cattle. Neg= below limit of detection (1.57 log_{10} GCN/ml). NA=sample not collected. '-'=Animal euthanized prior to this time point. 'post inoc.'=samples collected directly after inoculation

Non-Vaccinated Category I (pre-viremic/pre-clinical at time of euthanasia)

Animal ID	Sample type	Time point (hpi)									
		0	*post inoc.*	*4*	*6*	*8*	*10*	*12*	*24*	*48*	*72*
**1**	**Nasal swab**	Neg	5.34	6.11	4.87	3.44	3.76	4.54	-	-	-
	**Oral swab**	Neg	5.22	5.19	3.27	4.20	3.06	3.21	-	-	-
	**Serum**	Neg	*NA*	*NA*	*NA*	*NA*	*NA*	Neg	-	-	-
**2**	**Nasal swab**	Neg	7.81	5.90	3.79	3.34	3.66	3.93	-	-	-
	**Oral swab**	Neg	5.53	5.42	3.48	3.34	3.20	3.60	-	-	-
	**Serum**	Neg	*NA*	*NA*	*NA*	*NA*	*NA*	Neg	-	-	-
**3**	**Nasal swab**	Neg	5.72	*NA*	*NA*	*NA*	*NA*	*NA*	4.89	-	-
	**Oral swab**	Neg	5.86	*NA*	*NA*	*NA*	*NA*	*NA*	2.96	-	-
	**Serum**	Neg	*NA*	*NA*	*NA*	*NA*	*NA*	*NA*	Neg	-	-
**4**	**Nasal swab**	Neg	6.65	*NA*	*NA*	*NA*	*NA*	*NA*	5.79	6.63	-
	**Oral swab**	Neg	3.47	*NA*	*NA*	*NA*	*NA*	*NA*	3.62	4.11	-
	**Serum**	Neg	*NA*	*NA*	*NA*	*NA*	*NA*	*NA*	Neg	2.61*	-
**5**	**Nasal swab**	Neg	5.65	*NA*	*NA*	*NA*	*NA*	*NA*	4.48	5.01	6.59
	**Oral swab**	Neg	5.23	*NA*	*NA*	*NA*	*NA*	*NA*	3.35	3.61	6.91
	**Serum**	Neg	*NA*	*NA*	*NA*	*NA*	*NA*	*NA*	Neg	Neg	3.35*

*Serum samples positive by qRT-PCR but negative by virus isolation

Non-Vaccinated Category II (viremic/pre-clinical at time of euthanasia)

Animal ID	Sample type	Time point (hpi)					
		0	*post inoc.*	*24*	*48*	*72*	*96*
**6**	**Nasal swab**	Neg	6.71	4.52	5.49	6.83	6.82
	**Oral swab**	Neg	5.39	3.25	4.91	5.33	7.01
	**Serum**	Neg	*NA*	Neg	Neg	Neg	3.37
**7**	**Nasal swab**	Neg	6.86	5.58	-	-	-
	**Oral swab**	Neg	6.13	3.37	-	-	-
	**Serum**	Neg	*NA*	3.46	-	-	-

Non-Vaccinated Category III (viremic/clinical at time of euthanasia)

Animal ID	Sample type	Time point (hpi)					
		0	*post inoc.*	*24*	*48*	*72*	*96*
**8**	**Nasal swab**	Neg	8.03	5.43	3.90	-	-
	**Oral swab**	Neg	5.90	4.83	6.69	-	-
	**Serum**	Neg	*NA*	3.34	5.95	-	-
**9**	**Nasal swab**	Neg	6.03	Neg	4.45	8.30	-
	**Oral swab**	Neg	4.26	Neg	4.50	11.02	-
	**Serum**	Neg	*NA*	Neg	6.20	7.94	-
**10**	**Nasal swab**	Neg	6.51	5.52	5.84	7.24	7.02
	**Oral swab**	Neg	7.27	5.34	6.24	9.05	8.65
	**Serum**	Neg	*NA*	3.97	6.69	7.89	8.37

Vaccinated Cattle

Animal ID	Sample type	Time point (hpi)				
		0	*post inoc.*	*24*	*48*	*72*
**11**	**Nasal swab**	Neg	6.36	5.46	-	-
	**Oral swab**	Neg	5.08	5.21	-	-
	**Serum**	Neg	*NA*	Neg	-	-
**12**	**Nasal swab**	Neg	6.66	3.45	-	-
	**Oral swab**	Neg	5.14	Neg	-	-
	**Serum**	Neg	*NA*	Neg	-	-
**13**	**Nasal swab**	Neg	5.91	Neg	Neg	-
	**Oral swab**	Neg	5.11	Neg	Neg	-
	**Serum**	Neg	*NA*	Neg	Neg	-
**14**	**Nasal swab**	Neg	6.37	Neg	Neg	-
	**Oral swab**	Neg	5.03	Neg	Neg	-
	**Serum**	Neg	*NA*	Neg	Neg	-
**15**	**Nasal swab**	Neg	6.60	4.82	Neg	Neg
	**Oral swab**	Neg	Neg	4.56	Neg	Neg
	**Serum**	Neg	*NA*	Neg	Neg	Neg
**16**	**Nasal swab**	Neg	6.95	3.30	Neg	Neg
	**Oral swab**	Neg	5.69	Neg	Neg	Neg
	**Serum**	Neg	*NA*	Neg	Neg	Neg
